# Protonate3D: Assignment of ionization states and hydrogen coordinates to macromolecular structures

**DOI:** 10.1002/prot.22234

**Published:** 2008-09-02

**Authors:** Paul Labute

**Affiliations:** Chemical Computing Group Inc.Montreal, Quebec, Canada H3A 2R7

**Keywords:** protein titration, macromolecular protonation states, unary quadratic optimization

## Abstract

A new method, called Protonate3D, is presented for the automated prediction of hydrogen coordinates given the 3D coordinates of the heavy atoms of a macromolecular structure. Protonate3D considers side-chain “flip,” rotamer, tautomer, and ionization states of all chemical groups, ligands, and solvent, provided suitable templates are available in a parameter file. The energy model includes van der Waals, Coulomb, solvation, rotamer, tautomer, and titration effects. The results of computational validation experiments suggest that Protonate3D can accurately predict the location of hydrogen atoms in macromolecular structures. Proteins 2009. © 2008 Wiley-Liss, Inc.

## INTRODUCTION

As of October 2007, The protein data bank^1^ contained in excess of 45,000 structures, mostly the result of X-ray diffraction at resolution values greater than 1.5 Å. At these resolutions, the coordinates of hydrogen atoms cannot be observed and yet half of all atoms in the studied compounds are hydrogen atoms. Modern computational methodologies such as molecular mechanics, molecular dynamics, crystallographic refinement, electrostatic analysis, and docking require explicit (polar) hydrogen atoms for best results. Consequently, hydrogen atoms must be introduced into the X-ray structure data prior to undertaking these sorts of calculations. Thus, there is a need for automated procedures to predict hydrogen coordinates given the 3D coordinates of macromolecular structures.

The prediction of hydrogen coordinates from hydrogen suppressed macromolecular structures, say proteins, is nontrivial: (a) rotamers of hydroxyls, phenols, thiols, methyls, and primary amines must be determined; (b) the ionization states of acidic and basic groups, such as carboxylic acids, amines, guanidines, imidazoles, and possibly phenols and even alcohols, must be determined; (c) the ionization states of transition metals must be assigned; (d) the orientation of water molecules must be determined; and (e) the tautomeric state of imidazoles and other moieties must be determined. Additionally, it is common to include the determination of terminal group “flips.” A flip is a conformational difference or element identity exchange, say, in the terminal amide of asparagine or glutamine. Because of limited resolution, the identities of oxygen and nitrogen often cannot be reliably determined; consequently, there may be an ambiguity in PDB crystal structures. Flip ambiguities can also exist for the imidazole rings in histidine, terminal sulfonamides, and phosphonamides.

It is important to remember that protonation state prediction from static structures is motivated by the practical needs of molecular simulations or interpretation of structures. Inherently dynamic interactions of chemical groups with solvent (e.g., hydroxyl rotamers or weak acids and bases) cannot reasonably be expected to be predicted correctly in all cases. Instantaneous quantities, such as definite coordinates or ionization state, have little theoretical thermodynamic significance and, from a strict thermodynamic perspective, can be (at times) meaningless. In addition, coordinate errors in the nonhydrogen (heavy) atoms may result in unrealistic predictions; for example, unrealistically close crystallographic contacts exist in some structures. Notwithstanding these caveats, accurate prediction of protonation state and geometry has been the subject of much attention.

In 2005, Forrest *et al*. compared a number of programs^2^ that predict hydrogen coordinates given heavy atom structures: MCCE,^3^ CHARMM,^4^ CNS,^5^ GROMACS,^6^ Reduce,^7^ WHAT IF,^8^ and X-PLOR.^9^ Subsequent to the publication of the study, the ICDA procedure was published.^10^ We will not repeat the results of the Forrest study here; however, we will make some broad methodological comparisons in the interests of placing this work into the context of prior methods. The protonation state/geometry prediction methods can be classified broadly according to whether (a) the state space search is systematic or stochastic, (b) the energy/scoring function is physicochemical or geometric/heuristic, and (c) there is a formal attempt at titration free energy optimization.

GROMACS, CHARMM, and X-PLOR make no formal attempt to fully optimize the hydrogen interaction network. Discrete dihedral sampling and local force field-based interactions or a sequential, greedy buildup procedure is used in an effort to get a reasonable hydrogen placement. CNS places hydrogens at random and uses molecular dynamics and energy minimization to improve the configuration. MCCE uses a local systematic search followed by a Monte Carlo optimization procedure to place the hydrogens. A force field energy function is used for scoring with an optional solvation model (which dramatically increases the run time). The Reduce, WHAT IF, and ICDA programs employ a systematic search of torsion angles after first partitioning the system into clusters of atoms that are connected by potential hydrogen bonds. Reduce, WHAT IF, and ICDA depend critically on partitioning the system into small clusters each of which is subjected to a brute force optimization; for this reason, short interaction cutoffs on the order of hydrogen bond distances (3–4 Å) are required for tractable run times. Such short interaction cutoffs can be problematic for long-range ionic interactions, which fall off with the inverse of interionic distance; Reduce and WHAT IF use geometric/heuristic scoring and do not attempt formal titration and, consequently, this short cutoff assumption fits better with the approximate nature of the calculation. In contrast, ICDA uses an all-atom energy function with long-range Generalized Born electrostatics and yet a 3.1Å interaction cutoff appears to be used to partition the system into independent parts and long run times are suggested (20 min for 100 residues apparently on a 32 node cluster).

Other than the functional form of the underlying energetic model, the central problem to solve is the optimization of the hydrogen/ion interaction network. Even if a continuous rotamer model is used, the tautomeric and ionization state space is discrete. This leads to a combinatorial optimization problem similar to that of macromolecular titration calculations^11^,^12^ in which the interactions of titratable groups affect the individual propensities for protonation. The particular optimization problem is similar to that of side-chain conformation prediction in proteins. The conformational preference for a particular side chain is influenced by interactions with other side chains whose conformation is unknown. Programs such as SCWRL^13^ use graph theory to reduce the complexity of the calculation. Dead-end elimination^14^ is often used to eliminate energetically poor states. Monte Carlo,^15^ group clustering methods and partitioning methods,^10^,^11^ and other methods^16^,^17^ have also been used to efficiently search the configurations for an optimal energy configuration.

In this article, we present a new method—Protonate3D—which predicts hydrogen geometry, ionization, and tautomer states for macromolecular structures given the 3D coordinates of the nonhydrogen atoms. To our knowledge, Protonate3D is the first method that systematically optimizes the free energy of a macromolecular system in reasonable time, using a reasonably accurate electrostatic and implicit solvent model, and that takes longer range interactions into account, without partitioning the system into unrealistically small parts. We will describe the thermodynamic theory and a Unary Quadratic Optimization algorithm that obviates the need for unrealistically short interaction cutoffs and is the key to tractable run times. We will present the results of computational validation experiments and draw conclusions in the final section.

## THEORY AND METHODS

Consider a macromolecular system of *n* (nonhydrogen) atoms with Cartesian coordinates. To rapidly evaluate the energy of a particular configuration of the system (including hydrogens), we will decompose the system into a collection of distinct chemical groups, {*A*_*i*_}, consisting of atoms for which the protonation state is unknown and a set *P*, the part of the system for which there is assumed to be no uncertainty regarding its protonation state.

The decomposition proceeds as follows: implicitly break all bonds between 4-coordinated alkane sp^3^ carbon atoms and collect the resulting connected (bonded) groups of atoms. For proteins, this will leave the backbone intact, isolate the alkane carbons, and produce a collection of *m*-methylamide (Asn, Gln), thiomethanol (Cys), methylimidazoles (His), methylguanidinium (Arg), methyl carboxylic acids (Asp, Glu), methanol (Ser, Thr), indole (Trp), methylphenol (Tyr) and methylbenzene (Phe), methylamine (Lys), and thioether (Met) groups. A special case disconnection of the standard termini will produce a methyl amine (N terminus) and a methyl carboxylic acid (C terminus). Solvent and disconnected ions are considered to be separate groups. Collect the backbone and isolated alkane atoms into a set, *P*, the “known” portion of the system. The remaining atoms in the chemical groups are collected (by connectivity) into *m* sets, {*A*_*i*_}, the sets of the atoms for which there is uncertainty with respect to their protonation geometry, tautomer, or ionization state. This decomposition procedure assumes that alkane carbons and the protein peptide backbone have a known protonation state. In principle, any partitioning method can be used by Protonate3D provided that (relatively) apolar bonds are used to divide the system. The reason for this has to do with the thermodynamic approximations and the calculation of partial charges (which will be described later).

The hydrogen atoms of the heavy atoms of *P* (the “known” atoms) are added at standard bond lengths and angles according to the hybridization state of the atoms; for example, the backbone nitrogen in nonproline peptide bonds is given one hydrogen in the peptide plane; the C_α_ of nonglycine residues is given one hydrogen placed in an ideal tetrahedral geometry; sp^3^ carbons with two heavy neighbors (e.g., C_β_ of Glu) are given two hydrogens placed at ideal tetrahedral geometry; terminal methyls are given three hydrogens in tetrahedral geometry in staggered conformation with respect to their (necessarily) alkane carbon neighbors. Henceforth, *P* will denote the hydrogen augmented set of atoms in the “known” part of the macromolecule.

**Figure 1:**
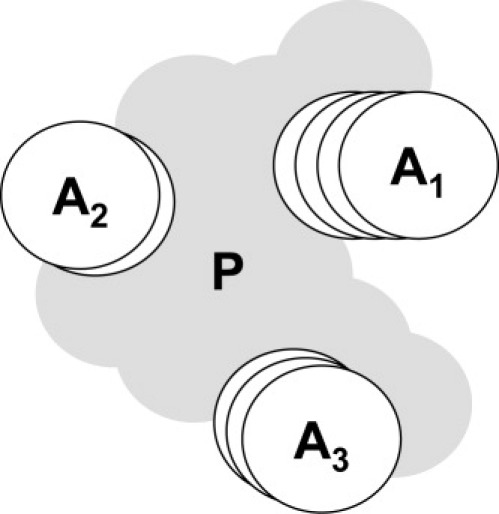
A diagram of a hypothetical macromolecular system. P consists of the atoms with known protonation state and geometry, A_1_, A_2_, and A_3_ denote chemical groups of atoms for which the protonation state is unknown; each group has a finite number of alternative states consisting of combinations of ionization, tautomer, flip, and rotamer configurations; in the diagram: four states for A_1_, two states for A_2_, and three states for A_3_.

For each chemical group *A*_*i*_, we generate a finite collection *S*_*i*_ = {*A*_*i*1_,*A*_*i*2_,…} of states consisting of the heavy atoms, flipped states, and all rotamer, tautomer, and ionization/protonation combinations of hydrogen atoms (see Fig. 1). In general, the states of chemical groups are generated according to a parameter file containing definitions of each chemical group and all of their topological tautomer and ionization states. The parameter file also contains, for each state, a tautomer strain energy (to provide for tautomer preferences). Rotamer (conformational) strain energy of each state is also considered and generated from force field parameter files such as OPLS-AA^18^ by applying the dihedral energy terms to the fragment geometry (as though still connected to *P*) and the intrafragment van der Waals energy terms (interfragment energies are handled by the matrix formulation of Eq. (1), later).

For proteins, the sp^3^ carbon atoms with two heavy neighbors are given hydrogens in a similar manner to the carbons of *P*; sp^2^ carbon atoms with one heavy neighbor (e.g., aromatic carbons) are given one hydrogen at standard bond lengths and angles in the π system plane. Primary amides are given two hydrogens at standard planar geometry; planar nitrogen atoms with two heavy neighbors and one hydrogen has that hydrogen placed in-plane at standard bond lengths and angles. The polar hydrogens and terminal methyls are given hydrogens appropriate to their ionization state and hybridization at standard bond lengths and angles. The dihedral combinations are determined according to the chemical type of the heavy atom: hydrogens in hydroxyls and thiols are sampled at 60° dihedral increments starting at a staggered rotamer; phenol hydrogens and other conjugated hydroxyls are sampled at 30° dihedral increments starting at an in-plane rotamer; methyls and primary amines are sampled at 60° dihedral increments starting at an extended conformation; hydrogens on other terminal atoms are given similar geometries. The anionic state of phenols, alcohols, thiols, and indoles are generated in addition to the neutral forms. The flip states of terminal amides, sulfonamides, and phosphonamides are generated. The anionic state and both neutral tautomers of carboxylic acids are generated (with the hydrogen cis to the carbonyl oxygen). Primary amines are generated in neutral and cationic forms and dihedral angles sampled at 60° increments starting at a staggered rotamer. Imidazoles are generated in anionic, cationic and two neutral tautomers (HID and HIE) as well as in flipped states (for a total of eight states). The states neutral of guanidines consist of all planar tautomers and rotamers. Water states consist of ∼500 rigid body orientations and isolated metals are given appropriate ionization states for groups I and II and a collection of ionization states from {+1,+2,+3} for transition metals under the assumption of zero ionization potential.

Thus, each *A*_*ij*_ consists of an all-atom chemical group with an appropriate ionization state, the heavy atoms, all of its hydrogen atoms in reasonable geometry and has an associated internal energy, *s*_*ij*_, consisting of the sum of its conformational and tautomeric energy. Figure 1 depicts a hypothetical fixed part *P* (with known protonation state and geometry) of a macromolecular system and three chemical groups each with a collection *S*_*i*_ of alternative protonation states; *A*_1_ has four alternative states, *A*_2_ has two states, and *A*_3_ has three states.

To represent the state ensemble of the system, arrange all of the individual chemical group states in all of the {*S*_*i*_} into single state list, *S*, divided into contiguous blocks corresponding to the {*S*_*i*_}, each of length *m*_*i*_ = |*S*_*i*_|.
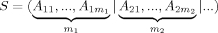


The first block of *m*_1_ elements in the list are the states of chemical group 1, the next block of *m*_2_ elements in the list are the states of group 2, and so on. (The reason for this arrangement will become clear shortly.) A configuration of the entire system consists of a selection of exactly one particular state from each block associated with a chemical group. Thus, there are a total of *m*_1_ × *m*_2_ × *m*_3_ × … configurations of the system. In typical proteins, the number of configurations exceeds 10^100^. A binary vector **x** of length equal to the length of the list *S* conveniently encodes a configuration, with a value 1 denoting the selection of an individual state. For example, in Figure 1, the vector **x** = (0,1,0,0,1,0,0,0,1) denotes the configuration state 2 from group 1, state 1 from group 2, and state 3 from group 3; to see this, introduce dividers into **x** corresponding to the blocks: **x** = (0,1,0,0 | 1,0 | 0,0,1), so that the position of the 1 value within each block (counting from the left) indicates the number of the state within the group. Admissible, or permitted, configuration vectors, **x**, have the property that there is exactly one 1 value in each block corresponding to a chemical group; this means that an admissible configuration vector encodes a definite single state for each chemical group. This constraint giving rise to the admissible configuration vectors is called the unary constraint, inspired by unary (base 1) notation of numbers in which “1” = 1, “10” = 2, “100” = 3, “1000” = 4, “10,000” = 5, and so on.

Suppose that we are given a pairwise interaction energy function *f*(*i*,*j*), for atoms *i* and *j* (e.g., Coulomb's law or a Lennard-Jones van der Waals potential), without loss of generality, we will assume that *f*(*i*,*i*) is well defined (e.g., for Coulomb's law, *f*(*i*,*i*) = 0). If *X* and *Y* are two disjoint sets of atoms (e.g., two chemical states), then the interaction energy between *X* and *Y* is
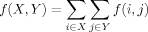


Form a matrix **U** with entries equal to the interaction energy of the various chemical group states in the list *S*. We will take the interaction energy between two states of the same chemical group to be zero. For notational convenience, let *I*(*k*) denote the chemical group to which state *k* belongs. Thus, the matrix **U** will have *U*_*ij*_ = *f*(*A*_*i*_,*B*_*j*_) if *I*(*i*) ≠ *I*(*j*) and 0 otherwise. Form a vector **u** with entries *u*_*i*_ = *f*(*P*,*A*_*i*_) + *s*_*i*_, the interaction energy between a chemical group state and the known part of the protein, *P*, and the internal energy of the state, *s*_*i*_ (to be described later). Let *u*_0_ = *f*(*P*,*P*)/2, the (constant) internal interaction energy of the known part of the protein *P*. With this matrix notation, we can write the total energy of a particular configuration encoded by admissible binary vector, **x**, compactly (and efficiently) with(1)
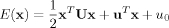


Thus, the total energy of a configuration of the system specified by **x** can be evaluated by a multidimensional quadratic form. If all of the values of **u** and **U** are calculated in advance, then a matrix–vector multiplication and two inner products are all that is required to evaluate the total energy for any arbitrary configuration of the system. Finding the optimal configuration of the system now is a matter of finding the smallest value of the quadratic form *E* over all binary vectors **x** satisfying the unary constraint; this optimization problem is called the “Unary Quadratic Optimization” problem.

Postponing the details of the energy model, the algorithmic structure of Protonate3D is (a more detailed set of steps is given at the end of this section):

1. Decompose the system by cutting apolar chemical bonds to determine the chemical groups, {*A*_*i*_}, and the “known” part of the system P.
2. Generate a collection of rotamer/tautomer/ionization states for each chemical group, *S*_*i*_ = {*A*_*ij*_}.
3. Calculate the values of the **U** matrix and the **u** vector as well as the constant *u*_0_ with a suitable energy model (to be described later); thus, configuration energies can be rapidly evaluated.
4. Find the binary vector **x** satisfying the unary constraint that minimizes Eq. (1); that is, solve the Unary quadratic optimization problem with a state-space search (see later).
5. Output the configuration encoded by *x*.

The addition of many (more than 20) water molecules (each with ∼500 orientations) becomes impractical. As a result, most of the water molecules are typically left out of the preceding steps and oriented afterward. This is done by orienting the waters one by one proceeding from the water in the strongest electrostatic field (of the protein and previously oriented waters) to the weakest. The selection of water molecules to include in the main calculation is left to the user—typically, water molecules near the sites of interest are treated in the main calculation.

The Unary quadratic optimization algorithm used by Protonate3D proceeds as follows. First, a dead-end elimination^14^ procedure is applied to eliminate states that cannot possibly be part of the optimal solution. This has the effect of reducing the dimensions of the **U** matrix and **u** vector of the quadratic energy function in a provably correct way. Suppose, elements *r* and *s* of the list *S* belong to the same chemical group *X*; if(2)
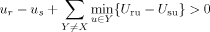
(where the sum extends over all chemical groups *Y* different from *X*) we can eliminate state *r*. The dead-end elimination criterion, when satisfied, eliminates *r* because no matter what state assignment is made, some state *X*, different from *r*, will result in a lower energy. This criterion is applied repeatedly until no more elimination is possible. Typically, the majority of the configurations are eliminated a priori, but it is still not practical to conduct a brute force search over the remaining configurations.

In an effort to speed up the state space search to follow, a “Mean Field Theory” calculation is performed to produce a Boltzmann distribution over all of the remaining individual chemical group states. This results in an estimate of the probability of each state in the Boltzmann-weighted ensemble of configurations. Briefly, the state probabilities **p**_*k*_ are determined by solving the nonlinear equation.(3)
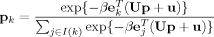
where **p** is the probability vector; **U** and **u** are as in Eq. (1); **e**_*k*_ is a vector of all zeros and a single 1 at position *k*; and β = 1/*kT*. The nonlinear equation can be solved efficiently by successive feedback iteration. These probabilities, **p,** are the population probabilities of the individual states under the assumption that each state feels the Boltzmann weighted average interactions of the other states. The vector **p** is used as a heuristic state priority in the subsequent search over states; the idea is to investigate high mean field probability states first under the assumption that they will lead to low energy configurations of the entire system (an approximate best-first search). The mean field probabilities, **p,** only affect the run-time of the state-space search and not its correctness; moreover, the energy of a system is evaluated using Eq. (1), which does not depend on **p**. The value of β must be chosen carefully to guarantee the uniqueness of **p**; in general, the solutions to Eq. (3) depend on the starting **p** vector. However, for certain values of β, the solution will be independent of the starting point (see the Appendix) and consequently **p** can be initialized with a uniform distribution on the states of each chemical group.

Finally, a recursive tree search is conducted over all admissible binary vectors, **x**, to locate the lowest energy state as calculated by Eq. (1) (which provides for rapid evaluation of energies). The performance of the search depends critically on the ability to prune the search space without loss of correctness. At any given point in the search, some of the elements of **x**, corresponding to some set of groups, *G*, will be assigned and others are yet to be assigned (with zero values). A lower bound, *L*(**x**), on the minimum energy of the system assuming the assigned part of **x** is
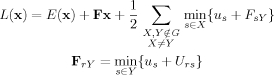


If this lower bound value exceeds the energy of the best energy determined thus far, then no further search of configurations containing the assigned part of **x** is required, thereby pruning the search tree and bypassing the examination of descendant configurations. During the recursive search, trial elements of the unassigned portion of **x** are made in decreasing order of the mean field probability. This greatly improves the pruning performance of the lower bound because the likelihood of visiting the best configurations first is increased. Moreover, premature termination of the search will produce the best solution with high probability.

**Figure 2:**
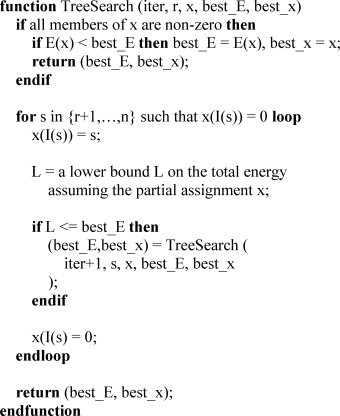
Pseudocode for the recursive state search (see text); the index *r* is the position in a mean field probability sorted list and *n* is the length of the list; *I*(*r*) denotes the chemical group corresponding to position *r*. The vector **x** holds the indices of the select' chemical group. The search is started with TreeSearch (0, [0,….,0], ∞, [0,…,0]).

The pseudocode for the recursive tree search procedure is given in Figure 2.

We now turn to the energy model for the macromolecular system. We will use an energy model that contains van der Waals repulsion, Coulomb electrostatic, and Generalized Born implicit solvation energies. Use of the Poisson-Boltzmann Equation (PBE) was not attempted because it was felt that the run-time would be prohibitively long for large systems, requiring at least one PBE solution per state. The van der Waals and Coulomb functional forms terms are pairwise and fit neatly into the quadratic form of Eq. (1); however, the Generalized Born model is not a two-body potential and certain approximations will be used to reformulate it into an effective two-body potential. In addition, because the number of particles may change upon ionizing a chemical group, we must introduce free energy terms related to group titration (because potential energies cannot be compared for systems with different numbers of particles).

Each atom of the system, whether in the known part, *P*, or in one of the group states {*A*_*ij*_} has associated van der Waals radius, van der Waals well depth parameters, as well as a partial charge. The van der Waals parameters and partial charges are permitted to depend on the particular tautomer, rotamer, or ionization state of each chemical group. In the interests of efficiency, we impose the requirement that the van der Waals parameters and partial charge assignments of one chemical group do not depend on the particular state selection of another chemical group. In particular, we require that the partial charge model be a nonpolarizable charge model (see the titration theory, later). The decomposition of the system along apolar bonds is done to reduce the potential adverse impact of these independence requirements.

Protonate3D uses a slightly modified version of MMFF94^19^ partial charges because (a) the MMFF94 charge model is based on fixed (topological) bond charge increments; (b) the chemical contexts for atom types in MMFF94 do not cross sp^3^ carbon atoms; (c) the bond charge increment between sp^3^ carbon bonds is zero (a purely apolar bond); and (d) MMFF94 supports general organic compounds. The slight modification to the MMFF94 charge model is that the normal zero bond charge increment between alkane hydrogens and carbons was replaced with a bond charge increment of 0.08 electrons, in better agreement with protein force field partial charges such as AMBER.^20^ Protonate3D uses Engh–Huber^21^ van der Waals parameters; however, hydrogens on oxygen and nitrogen are taken to have zero van der Waals radius, consistent with OPLS-AA. Coulomb's law is used for electrostatic interactions and special form of van der Waals interaction is used: only the repulsive part of the van der Waals interaction energy is modeled (although, the standard Lennard-Jones functions with the attractive term are not precluded). The special functional form is 800ε_*ij*_ (1 − *r*/*R*_*ij*_),^3^ where *r* < *R*_*ij*_ is the interatomic separation, *R*_*ij*_ is the sum of the van der Waals radii, and ε_*ij*_ is the geometric mean of the van der Waals well depth parameters for the two interacting atoms. Because of the 800 factor derived from a series expansion, this functional form lies in between the 12-6 and 9-6 Lennard-Jones functions at distances below the optimal interaction distance and approximates the 12-6 form well near the energy minimum. Because the OPLS-AA van der Waals parameters for polar hydrogen atoms are zero, the van der Waals terms are used by Protonate3D to handle side-chain “flip” states; the special form was used largely to mimic the sphere overlap test of Reduce.^7^ The elements of **U** matrix and **u** vector are populated by a straightforward application of the pairwise formulae given previously.

Protonate3D uses a modified version of the Generalized Born/Volume Integral (GB/VI) formalism^22^ for implicit solvent electrostatics (although other Generalized Born models are not precluded):(5)
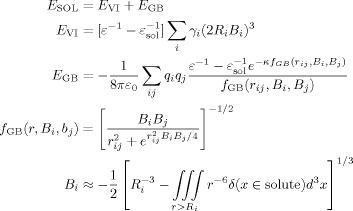


In this equation, ε is the dielectric constant of the interior of a solute, ε_sol_ is the dielectric constant of the solvent, {γ_*i*_} are (topological) atom-type-dependent constants that account for nonpolar energies including cavitation and dispersion using an inverse sixth-power integral instead of surface area, {*R*_*i*_} are (topological) atom-type-dependent solvation radii, κ is the Debye ionic screening parameter that depends on salt concentration, {*q*_*i*_} are the atomic partial charges, {*B*_*i*_} are the Born self-energies (inversely proportional to the Born radii), which are estimated with a pairwise sphere approximation^23^ to the solute cavity, and *r*_*ij*_ denotes the distance between atoms *i* and *j*. Were it not for the {*B*_*i*_}, the GB/VI equations would be a pairwise potential; however, because the *B*_*i*_ of a particular atom *i* depends on the state assignment of atoms in other chemical groups with possibly unknown state, we must calculate a set of {*B*_*i*_} that (a) remain fixed despite the protonation state of other groups and (b) reasonably preserve the GB/VI energy values.

Consider an atom *k* in the system (whether in *P* or in some state *A*_*ij*_). The contribution to *B*_*k*_ from all of the other atoms in the system will fall as the sixth power in the integrand of Eq. (7). Thus, atoms far away from *k* will contribute little, no matter if they are in some other group with unknown state. The various states in the system differ only in the position or absence of hydrogen atoms, which contribute relatively little to the volume integral (because of their small solvation radius); thus, the bulk of the states' contribution (from the heavy atoms) will be accurate no matter which state is selected. In any event, the approximation to the volume integral in the GB/VI is a pairwise summation of the form
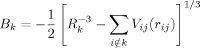
for a specific function^22^ *V*. To minimize the impact of the hydrogen positions of the unknown states, Protonate3D uses a separate mean field approximation to the volume integrals. A separate **U** matrix and **u** vector is created containing only the van der Waals repulsion terms, the states' internal strain energies, and the pH-dependent isolated group titration energies (see later). For each separate group state, the mean field equation of Eq. (3) is then solved to produce a set of state probabilities **p**. Each atom in each group state as well as the known part *P* is given the probability of its chemical group state, or 1 if the atom is in *P*. The Born factors are then calculated with(6)
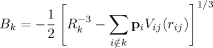
resulting in a mean field approximation to the Born factors that takes steric, rotamer/tautomer, and isolated group p*K*_a_ free energies into account. This approximation works very well in practice; indeed, one can argue it is in some sense superior to the original in that it takes some protonation state flexibility into account. It should be noted that some GB implicit solvent models do not include hydrogens in the volume integration^24^; consequently, we believe that our calculation of the mean field Born factors is eminently reasonable. In this way, we approximate the three-body GB/VI model with a close pairwise model more suited for the quadratic form of Eq. (1).

It remains to deal with the pH-dependent free energy of ionization of the chemical groups that must be included in the calculation. Consider the free energy, *a*, of the reaction PAH → PA^−^ + H^+^, where AH is an acidic group bound (possibly covalently) to a macromolecule P. Our approach is to introduce a thermodynamic cycle linking the reaction to the isolated group reaction AH → A^−^ + H^+^, whose free energy will be assumed to be known. In the covalent case, we consider the thermodynamic cycle
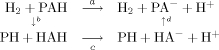
in which *a* = *b* + *c* + *d*. If the p*K*_a_ of the reaction HAH → HA^−^ + H^+^ is known (say from experiment), then for a given pH, we have that *c* = −*kT* (log 10) (pH−p*K*_a_), where *k* is Boltzmann's constant and *T* is the temperature of the system. Because the (vertical) reaction equation H_2_ + PAH → PH + HAH is balanced and, by construction, *E* = *E*_COUL_ + *E*_SOL_ is the free energy of charging and solvating the system, we may simply write
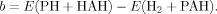


The case of a noncovalently bound group AH near a macromolecule P is simpler in that the H_2_ molecule is not required to balance the equation and, in this case,
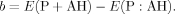


We shall deal with the noncovalent case first, because it is simpler and provides insight into the covalent case. The noncovalent *d* is similar to *b* resulting in
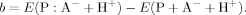
and using the fact that *E*(A + B) = *E*(A) + *E*(B) we have that(7)
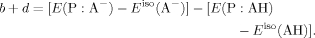


The superscript iso is used to signify that the *E* is calculated for the isolated AH and A^−^ systems (i.e., calculated with Born factors derived from the isolated system, ignoring *P*). These iso superscripted quantities involve only a small number of atoms—the atoms of AH and A^−^—and direct evaluations of *E* are used to calculate the required energy. The iso superscripted quantities are included directly in the **u** vector of Eq. (1) for the corresponding group state so that *b* + *d* is simply a difference of configuration energies.

With a similar line of reasoning as in the noncovalent case, we find that as a result of cancellations of *E*(PH) and *E*(H_2_), for the covalent case(8)
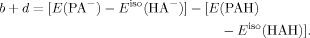
and, as before, the iso superscripted quantities can be calculated directly (because few atoms are involved) and included in the **u** vector. In practice, the distinction between covalent and noncovalent groups makes only a small difference—on the order of 0.5 kcal/mol (∼2% error) for ionic species. A small correction to the experimental isolated p*K*_a_ values for covalently bound species can account for most of this difference. In any event, the static nature of the entire calculation and the approximations inherent in a Generalized Born model will in all likelihood overshadow any lack of distinction between the cases.

The free energy *c* = −*kT* (log 10) (pH − p*K*_a_) remains to be included in Eq. (1). Consider a polyprotic species AH_*n*_ with p*K*_a_ values p*K*_*i*_, corresponding to AH_*i*_ → AH_*i*−1_. The free energy of the reaction AH_*i*_ → AH_*i*−1_ + H^+^ is then Δ*G*_*i*_ = −*kT* log 10 (pH − p*K*_*i*_). If we assign(9)
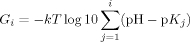
we will have that Δ*G*_*i*_ = *G*_*i*_ − *G*_*i*−1_; thus, we can incorporate the *G*_*i*_ values into the relevant **u** vector entries for each acidic chemical group state with *i* titratable protons. The reasoning for the *b* and *d* quantities generalizes to polyprotic species and multiple-site titration straightforwardly, because of the overall pairwise nature of the energy terms that make up the effective configuration energy.

We now summarize the Protonate3D procedure:

1. Decompose the system by cutting apolar chemical bonds to determine the chemical groups, {*A*_*i*_}, and the known part *P* (to which hydrogens are added in standard geometry).
2. Generate a collection of rotamer/tautomer/ionization states for all the chemical groups, *S* = {*A*_*ij*_} each with an associated internal strain energy, *s*_*ij*_.
3. Populate the **U** matrix and **u** vector values for the van der Waals repulsion, state strain *s*_*ij*_ energies, and the isolated titration free energies from Eq. (9).
4. For each group state in {*A*_*ij*_}, solve Eq. (3) for the mean field probabilities and use these probabilities to calculate the Born factors using Eq. (6).
5. Use MMFF94 to calculate partial charges for *P* and each of the {*A*_*ij*_}.
6. Add additional **U** matrix and **u** vector values for the Coulomb and GB/VI energy terms and the isolated group energy terms from Eqs. (7) and (8). Calculate *u*_0_, the internal Coulomb, GB/VI, and van der Waals energy for *P*.
7. Use the dead-end elimination criterion of Eq. (2) repeatedly to eliminate states that cannot be part of the optimal solution.
8. Solve Eq. (3) for the mean field probabilities (using the updated **U** and **u**) to order the list of states for the state search procedure.
9. Use the algorithm of Figure 2 to recursively search the configuration space, ordered by the mean field probabilities of step 8, pruning the search tree with Eq. (4), and determine the set of states that minimizes the quadratic energy function of Eq. (1).
10. Orient any incidental waters (not included in the main calculation) one by one starting from the water in the strongest electrostatic location of the energy minimum configuration, proceeding to the weakest, at each step taking into account previously oriented water molecules.

This brings to a close the exposition of the Protonate3D methodology. Protonate3D was implemented in the Scientific Vector Language of the Molecular Operating Environment^25^ version 2006.08. Computational experiments were conducted on a 2 GHz Pentium IV processor running Microsoft Windows.

## RESULTS AND DISCUSSION

The validation of a proposed macromolecular protonation procedure is a nontrivial undertaking and not always attempted.^7^,^10^ Ideally, one would find a definitive collection of 3D macromolecular structures containing hydrogen atoms and attempt a reconstruction of the hydrogen positions and ionization states using only the heavy atoms. Unfortunately, such a collection does not exist. Although the protein data bank contains a number of ultra-high resolution crystal structures, the diffraction resolution and the refinement procedures in common use cast some doubt upon the notion that the hydrogens were actually observed or reliably inferred.^2^ Notwithstanding these concerns, the PDB is still a good source of validation data, albeit for only a small fraction of the deposited structures.

It is tempting to think that experimental protein titration curves could be used to validate a protonation procedure. Neglecting the fact that a protonation procedure may not formally calculate a titration curve, such curves are indirect forms of validation. One problem lies with weakly acidic or basic groups: a group that is 50% protonated is not well represented by any single protonation assignment. More generally, it is an average of protonation states that produces the titration curve, not any single instantaneous or partially thermodynamic 3D state, which is what a protonation procedure produces as output.

We have chosen to validate Protonate3D by (a cautious) comparison of its output to ultra-high resolution crystal structures. Despite the problems with PDB depositions, we feel that X-ray crystal structures are the closest to direct experimental proton geometry observation. There are competing goals in the selection of a validation set: (a) comparison to other programs is desirable; (b) confidence in the hydrogen atom coordinates is desirable; and (c) nontrivial ionization cases should be present. We decided that confidence in the hydrogen atom geometry was paramount for our validation of Protonate3D, and we selected our structures on the basis of ultra-high resolution. Unfortunately, on such a validation set, there is little opportunity to compare Protonate3D with other programs or on challenging ionization examples from published accounts. We will not comment on how all of the other programs would fare on our validation set and leave this to a future study. However, we will examine a few nontrivial ionization cases using lower resolution PDB structures and comment upon what can be expected from the calculated protonation states and the relationship to experimental residue p*K*a values.

A search of the Protein Data Bank was conducted for all X-ray crystal structures with a resolution of 0.85 Å or better. Of these 52 structures, 30 contained no hydrogen coordinates, one (1X6Z) contained evidently dubious hydrogen coordinates with all of the H_2_O molecules oriented identically, and one (2IZQ) was a 15-mer antibiotic with alternate side-chain conformations for most of the residues. From the remaining 20 structures, redundancies were removed (e.g., multiple structures of trypsin) with the best resolution or most recent deposit retained. The PDB codes of the resulting nine nonredundant ultra-high resolution set of structures is shown in Table I along with the resolution, macromolecular chain length, crystallization pH, and the molecular entity contained in the deposit. Water molecules and small neutral solvent molecules (such as glycerol) were removed. (It was determined, by inspection and trial calculations, that the solvent removal would not appreciably affect the hydrogen assignments reported herein.) Ions, salts, and metals were retained in the structures. These prepared nine structures will be referred to, henceforth, as the “test collection.”

**Table I:** X-Ray Crystal Structures Used for Validation and Overall Atomic Agreement

Code^a^	Res^b^ (Å)	Len^c^	pH^d^	Compound	Time^e^ (s)	Atoms^f^	Agree^g^ (%)
1EJG^26^	0.54	46	7.0	Crambin	0.9	31	87
1GCI^27^	0.78	269	5.9	Subtilisin	11.3	185	99
1GDN^28^	0.81	242	6.0	Trypsin	19.0	167	85
1P9G^29^	0.84	41	5.5	Antifungal protein	0.9	20	90
1UCS^30^	0.62	64	7.5	Antifreeze protein RD1	1.3	76	91
1YK4^31^	0.69	53	6.0	Rubredoxin	1.5	56	88
2B97^32^	0.75	140	7.4	Hydrophobin II	2.2	128	89
2H5C^33^	0.82	198	4.3	Alphalytic protease	9.0	163	85
3PYP^34^	0.85	125	4.8	Photoactive yellow protein	4.1	119	95

a Superscripts denote manuscript references.

b Resolution (Å) of the X-ray diffraction.

c Number of residues of the main macromolecular chain.

d pH is taken from the PDB header (crystallization conditions).

e Run time of Protonate3D in seconds on a 2 GHz Pentium IV.

f Number of –;OH, –SH, –NH_i_, –CH_3_, –CO_2_, N(his) atoms with hydrogen occupancy ≥0.8.

g The percentage agreement of hydrogen placement to within 15° dihedral angle of experiment.

For each structure in the test collection, Protonate3D was run at 300 K with a 0.1 m*M* salt concentration and at the pH of crystallization conditions. A 15-Å cutoff was used for all nonbonded interactions. (Larger cutoffs were investigated and showed no significant differences.) The total time taken for each calculation (on a 2 GHz Pentium IV) is given in Table I. The longest calculation was for 1GDN, at 19 s, and the shortest was for 1EJG and 1P9G, at 0.9 s. The Protonate3D results were then compared to the deposited coordinates. Except where noted, hydrogen coordinates in deposited structures were only considered if the occupancy values were 0.8 or greater; for example, if a hydroxyl or methyl hydrogen had an occupancy value below 0.8, then the hydroxyl or methyl was not considered sufficiently resolved and the comparison with Protonate3D was not performed. Similarly, atoms all of whose hydrogens were missing (e.g., alkane carbon atoms) were not included in the comparison. Table I gives the percentage agreement to within 15° dihedral angle of the deposited coordinates of –OH, –SH, –NH_*i*_, –CH_3_, –CO_2_, N(histidine) atoms with an H occupancy ≥0.8. The lowest agreement was 85% and the highest was 99% (mainly because of low occupancy values of hydroxyl hydrogens). The total per-atom 15° dihedral angle agreement on the whole test collection was 90%.

### Nonrotameric hydrogen placement

Protonate3D places hydrogen atoms for many groups at standard geometries; for example, secondary and tertiary sp^3^ carbons (C_α_, –CH_2_–, etc.), aromatic carbons (Phe, Tyr, Trp, etc.), amide, peptide, guanidinium (Arg), and pyrrole (Trp) nitrogens. In the test collection, the hydrogen atoms were placed to within 0.2 Å of the deposited coordinates with almost 100% accuracy, where such hydrogens were deposited and had occupancies of at least 0.8. The two or three (out of several thousand) cases where Protonate3D deviated from this accuracy were clearly anomalous poor hydrogen geometries in the crystal structure deposition. Atoms for which there are generally no hydrogens (such as carbonyl oxygen in amides and peptides, disulfide bridges, and thioethers) are not protonated by Protonate3D and are therefore, in agreement with the structures in the test collection.

### Thiol rotamers

The test collection was chosen on the basis of resolution and the presence of hydrogen atoms in the deposited structure. Thiols with occupancies at least 0.8 (in Cys) were underrepresented with only two instances (1GDN) in the test collection. Four thiols with occupancies less than 0.8 of them were in 1YK4 and all were ligated to an iron atom in tetrahedral geometry. In 1YK4, Protonate3D assigned anionic states to Cys6, Cys9, Cys39, and Cys42, whereas the deposited structures were neutral with attached hydrogens. The (isolated) iron atom was assigned a +2 formal charge giving an overall −2 charge for the iron–sulfur group. In the deposited coordinates, the thiol hydrogen of Cys9 points directly at the backbone H of Tyr1 1.18 Å, and the thiol hydrogen of Cys42 interacts with the backbone H of Ala44 1.73 Å away. In both cases, the backbone hydrogens were buried and were it not for the thiol hydrogens, the backbone hydrogens would have made favorable polar interactions with the sulfur lone pairs. Thus, the deposited thiol coordinates are potentially in error for Cys9 and Cys42. The deposited thiol coordinates for Cys6 and Cys39 are directed toward more hydrophobic environments and so there is no clear inconsistency. In 1GDN, Protonate3D failed to agree with the two thiol rotamers to within a 15° dihedral angle. However, the sulfurs of Cys42 and Cys58 are 3.19 Å apart suggesting a possible disulfide bridge; however, in 1FN8, 1FY4, and 1FY5 (a 0.81 Å crystal structures of the same protein), Cys42 and Cys58 contain no hydrogen atoms but are not bonded in a disulfide bridge. The aforementioned structures were the only ones with resolutions under 0.85 Å containing apparent thiol groups, so we cannot draw any definitive conclusions about thiol rotamer accuracy.

### Hydroxyl/phenol rotamers

In the test collection, there were a total of 128 hydroxyl (Ser, Thr) and phenol (Tyr) groups with deposited hydrogen coordinates with occupancies at least 0.8. Hydroxyl and phenol groups are generally challenging because of the low rotational barriers, significant temperature factors, tendency for exposure to solvent, and multiple plausible polar interactions with their environment.^2^ Protonate3D placed –OH hydrogens to within a 15° dihedral angle of the deposited coordinates in 37% of the cases. Increasing the success criterion to a 50° dihedral angle resulted in a 46% success rate; in other words, when there was a disagreement it was quite large. There was no appreciable difference in accuracy between hydroxyls and phenols, nor was their any appreciable difference in accuracy that depended on the solvent exposure of the –OH group. Even if an MMFF94 energy minimization is conducted after the Protonate3D placement, with heavy atoms fixed, the success rate does not improve and the hydrogen coordinates do not change appreciably (results not shown). The inclusion of crystallographic waters near –OH groups did not change the overall accuracy results appreciably (results not shown). Of the groups that did not pass the 15° dihedral angle success test, 31% of them were positioned by Protonte3D in clearly better hydrogen bonding arrangements (as determined by distance and angle criteria between the hydrogens and nearby lone pairs) than the deposited coordinates whereas only 7% were placed in apparently worse arrangements. It should be noted that crystallographic images were not included in the calculation and it is possible that with their inclusion the results may change somewhat. Excluding the cases where the Protonate3D placement was deemed superior to the deposited coordinates, we have that in 45% of the cases Protonate3D agrees with the deposited coordinates to within a 15° dihedral angle. Including even cases where the occupancy was less than 0.8 did not change the accuracy results appreciably. The foregoing results suggest that (a) Protonate3D places hydroxyl/phenol hydrogens in credible low energy wells, (b) Protonate3D agrees with high-resolution crystal structures in 37% of the cases to within a 15° dihedral angle, (c) either there is a deficiency in the energy models used by force fields and Protonate3D or many of the hydrogen coordinates in hydroxyls and phenols in high-resolution structures may not be reliable, or both.

### Methyl rotamers

Protonate3D places hydrogen atoms on alkane methyl groups (Val, Ile, Leu, etc.) in staggered conformation and methyl groups in thioethers (Met) at either staggered or eclipsed conformations with a 0.5 kcal/mol conformational strain for eclipsed. In the test collection, 618 of 623 (99%) methyl groups with hydrogen occupancies at least 0.8 were positioned within 15° dihedral angle of the deposited coordinates. All five cases of disagreement had dihedral angle disagreements of less than 19°, narrowly missing the 15° success cutoff. These results suggest that the staggered conformations for alkane methyls used by Protonate3D are excellent predictors of high-resolution crystal structure methyl conformations.

### Primary amine rotamers

The primary amines of (Lys and N-termini) are placed in staggered conformation by Protonate3D (similar to alkane methyl rotamers). In the test collection, 24 of 26 (92%) primary amines with hydrogen occupancies of at least 0.8 were in 15° dihedral angle agreement with the deposited coordinates. In 1YK4 one disagreement was 22° and in 1UCS, the rotamer selected by Protonate3D had more favorable interactions and less strain (the deposited coordinates were in eclipsed conformation and very exposed to solvent). These results suggest that the staggered conformation used by Protonate3D is an excellent predictor of the conformations of primary amines in high-resolution crystal structures.

### Terminal amide and imidazole flips

Protonate3D uses electrostatic and van der Waals energies to assess the side-chain conformations of terminal amides and imidazoles (Asn/Gln/His) without any bias toward the deposited element identities. It is the remaining atoms of the system that ultimately determine the selected conformations. To assess the accuracy of the Protonate3D prediction, we compared the output element identities of Protonate3D with the deposited element identities in the test collection; success or failure was determined by element agreement on an entire group. Table II presents the results of the comparison. For each group, the percent correct predictions are listed along with the total number of instances in parenthesis. Accuracy was 88% for asparagine, 82% for glutamine, and 100% for histidine side chains. In the test collection, there were six cases of terminal amides that appeared to be in error because of unambiguous (to the eye) more favorable interactions in the flipped state and unambiguous (to the eye) unfavorable interactions in the deposited coordinates: 1GCI:Asn117, 1GCI:Gln182; 1GDN:Gln192, 1UCS:Asn1, 2B97:Asn10, 3PYP:Gln32. Leaving these out changed the results relatively little giving adjusted accuracies of 92% for asparagines and 86% for glutamine. In all cases, the terminal amide flips that were in disagreement were quite exposed to solvent with no unambiguous favorable interactions in proximity (for either flip state). No disagreements with terminal imidazole groups in histidine were observed. These results suggest that Protonate3D can determine the conformation of terminal amides and imidazoles with high probability.

**Table II:** Agreement of Side-Chain Flip Assignments

	ASN (%)	GLN (%)	HIS (%)
1ejg	100 (3)^a^		
1gci	95 (22)	70 (10)	100 (7)
1gdn	75 (12)	80 (5)	100 (2)
1p9g	100 (2)	100 (2)	
1ucs	80 (5)	100 (2)	
1yk4	100 (1)		
2b97	75 (4)	100 (6)	100 (2)
2h5c	92 (13)	89 (9)	100 (1)
2pyp	83 (6)	80 (5)	100 (2)
Total	88 (68)	82 (38)	100 (14)

a Parenthesized values are the total number of instances.

### Ionization states

Protonate3D formally calculates optimal ionization states for all polar atoms excepting the protein backbone. With the exception of the four Cys sulfurs ligated to the iron in 1YK4 and His103 in 3PYP, there was 100% consistency between the calculated side-chain ionization states and the test collection (several hundreds of cases). In other words, the hydrogen counts in the deposited structures were in agreement with the output of Protonate3D. (Histidines for which no polar hydrogens were submitted were omitted from the consistency calculation.) This statement should be treated with some caution; for example, a terminal CO_2_ group in a crystal structure without attached hydrogens can be interpreted as carboxylate or carboxylic acid but without observed hydrogens—the presence of hydrogens is an observation but the absence is not. We have observed 100% agreement on CO_2_ groups in the test collection (excepting 3PYP:Glu46 as described later), which suggests that Protonate3D does not add protons to CO_2_ groups very often (and neither do high-resolution crystallographers). Similarly, the traditionally neutral side chains of Asn, Gln, Ser, Thr, Trp, Tyr, and so forth were not ionized by Protonate3D, and the traditionally positive side chains of Lys and Arg were ionized by Protonate3D. These results support the assertion that Protonate3D does not generally assign unusual ionization states even though it is formally allowing them to compete in its energy optimization. It must be remembered, however, that pH range of the proteins in the (rather small) ultra-high resolution validation set was limited and did not contain sufficient dynamic range to fully test the ionization state assignments of Asp, Glu, Lys, and Arg. Moreover, a “null hypothesis model” of using the isolated group p*K*_a_ values would produce a similar prediction accuracy. Interestingly, we found that the protonation assignments to be less sensitive to the input pH than one might imagine, although this was not studied systematically. We speculate that the atomic coordinates of a group's environment are a more decisive factor in determining the assigned ionization state, at least in the context of using crystal structure coordinates as input.

**Figure 3:**
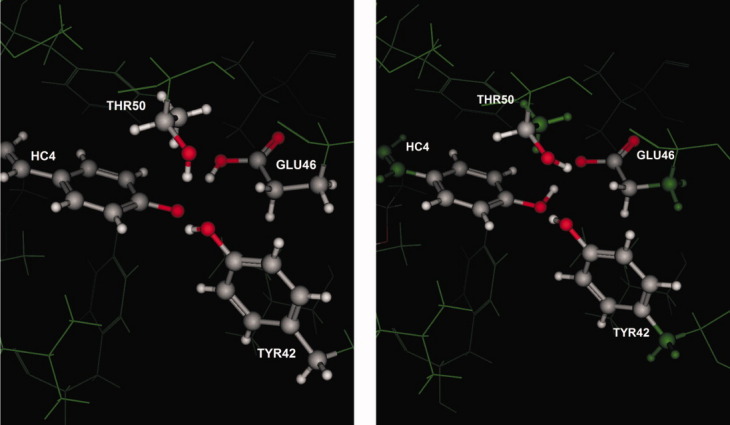
Left is a depiction of the deposited coordinates of 3PYP (photoactive yellow protein) in which Glu46 is neutral and in an unusual conformation and the phenol oxygen of the covalently bound ligand HC4 is apparently anionic. Right is a depiction of the Protonate3D protonation calculation in which the phenol is neutral and the Glu46 is anionic; the hydroxyl of Thr50 is rotated to donate its hydrogen in a polar interaction with the carboxylate.

Glu46 in 3PYP is an interesting case and the single case of a deposited neutral carboxylic acid in the test collection (and in all the 0.85 Å or better resolution crystal structures). The hydrogen on the carboxylic acid has an occupancy of 0.76, narrowly missing our usual 0.8 cutoff value. The deposited protonation state is depicted in Figure 3 (left); the Glu46 is neutral but in a strained trans conformation donating its hydrogen to an apparent anionic phenol on the covalently bound ligand denoted by HC4, Thr42, and Thr50 also donate their hydrogens to the anionic phenol oxygen in a loose tetrahedral geometry. Figure 3 (right) depicts the Protonate3D calculation results; here, the Glu46 is anionic and the nearby phenol is neutral. The hydroxyl of Thr50 is rotated and interacts favorably with the carboxylate. On the whole, the Protonate3D results seem more reasonable: the p*K*_a_ difference between carboxylic acid and phenol and the close proximity of the groups strongly favor the neutral phenol and anionic carboxylate configuration. These results suggest that the deposited coordinates may be in error or that the level of theory of Protonate3D is insufficient.

The histidine residues (with an isolated p*K*_a_ of ∼7) afford more of an opportunity to validate the ionization assignment of Protonate3D. In the test collection, there were no histidines near transition metals; consequently, we omitted histidines for which there were no (zero) polar hydrogens deposited on the imidazole ring. In total, there were 10 histidines for which hydrogen coordinates were deposited and where the polar protons had occupancies greater than 0.8. We proceeded under the assumption that the absence of a polar imidazole hydrogen indicated neutral group. We observed that eight of the 10 histidine side chains (80%) had ionization state and tautomer state assigned by Protonate3D in complete agreement with the deposited coordinates. In two cases, 1GCI:His64 and 3PYP:His108, there was a protonation state disagreement. For 1GCI:His64, Protonate3D predicted a cationic imidazole ring whereas the deposited structure assigned a neutral N_δ_ tautomer. In fact, the N_δ_ is pointing directly at a carboxylate oxygen of the buried Asp32, 2.64 Å away, which suggests an error in the deposited structure which we believe should have contained a cationic His64 to form a salt bridge with Asp32. For 3PYP:His108, Protonate3D predicted a neutral N_ε_ tautomer whereas the deposited coordinates contained a cationic imidazole. The immediate environment of His108 in the deposited structure is
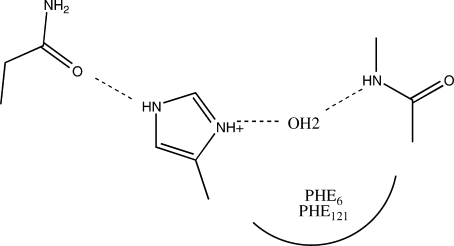


His108 is interacting with the terminal amide oxygen of Asn89 and the backbone nitrogen of Gly7 mediated by a water molecule. The water molecule is flanked obliquely by benzene rings of Phe6 and Phe121 and has no other hydrogen bonding opportunities. There is no obvious salt bridge interaction with His108. Protonate3D flipped neither the terminal amide of Asn89 nor the imidazole of His108. Consequently, the hydrogen on N_ε_ is confirmed reasonably; similarly, the backbone nitrogen of Gly7 clearly donates its hydrogen bond to the mediating water. Because there are no other hydrogen bonding opportunities for the water, the protonation state assignment depends on the water configuration and the ionization state of the imidazole. Because there is no clear salt bridge opportunity, the Protonate3D assignment of a neutral imidazole seems more reasonable, especially considering the relatively buried nature of the imidazole ring. In the Protonate3D assignment, the water molecule donates a hydrogen bond to the lone pair of N_δ_ on the neutral imidazole ring. In this way, the water molecule has only one hydrogen not participating in a hydrogen bond, whereas in the deposited coordinates the water has two hydrogens not participating in hydrogen bonds. It seems reasonable that the deposited coordinates for His108 are possibly in error. The foregoing results suggest that Protonate3D can predict the ionization and tautomer state of histidines in high-resolution crystal structures with high probability.

### Transition metal complexes

Unless specific entries in Protonate3D's parameter file are available, Protonate3D normally disconnects transition metals from their ligands. The transition metals are modeled as isolate point charges taking on values of {+1,+2,+3}, depending on the transition metal, with a zero ionization potential. Thus, the state of the transition metal is determined by its environment. In proteins, transition metals are typically complexed with histidines, carboxylates, and thiols. Protonate3D considers anionic species of these residues, which are relevant for transition metal complexes. Although this transition metal model is simplistic, we expect, nevertheless, that it will take into account the gross classical electrostatic effects that determine the protonation state of residues near the metal.

**Figure 4:**
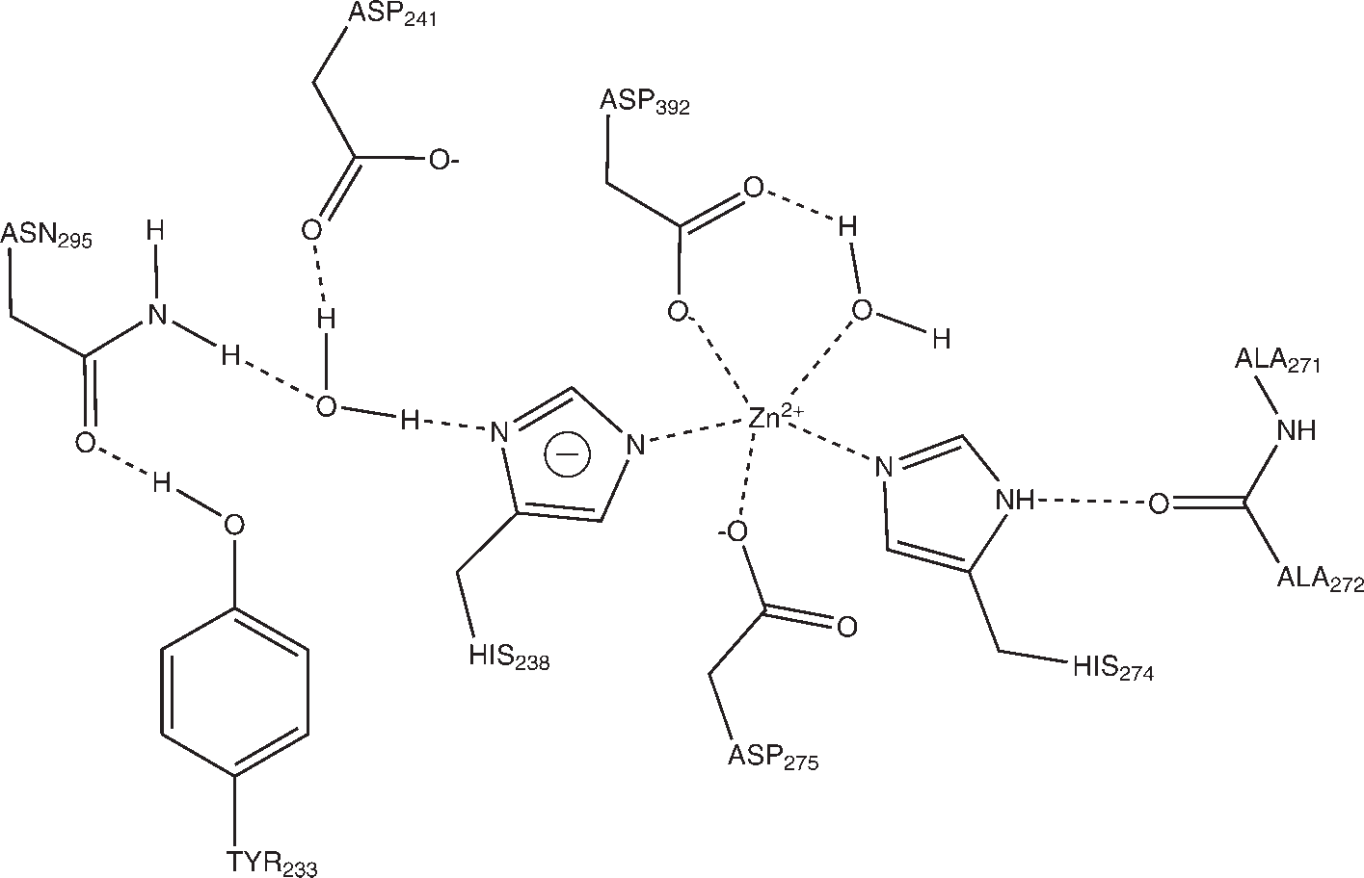
Protonate3D assignment of the protonation state of residues and waters around the zinc in phosphodiesterase IV (PDB:1RO6) complexed with rolipram.

To illustrate the use of Protonate3D near a transition metal complex, we will use the phosphodiesterase IV complex with rolipram found in PDB code 1RO6. This is 2.0 Å X-ray crystal structure of two 378 residue proteins (a homodimer) each complexed with rolipram. There is a 5-coordinated zinc in the active site with two histidines, two carboxylates, and a water as ligands. Adjacent to the metal complex, there is a backbone carbonyl, one water molecule, a terminal amide, and a phenol group. Figure 4 is a 2D diagram of the aforementioned residues. This small portion of the phosphodiesterase active site is a challenging environment for the prediction of protonation states, mainly because of the transition metal and water mediated interactions. Formally, the two histidines have eight possible states (including “flip” states), the terminal amide has two states (one “flip”), the phenol has a rotatable hydrogen, the carboxylates each have three states, and the water molecules have full rotational freedom. The backbone carbonyl of Ala272 forms a hydrogen bond with His274 and we can reasonably eliminate all but the N_δ_ neutral form for His274. One might also argue that the carboxylates of Asp275 and Asp293 are most likely anionic (although one must always be careful not to be too hasty with transition metal complexes). The real issues are the ionization state of His238, the rotamer of Tyr233, the “flip” state of Asn295, and the orientations of the water molecules.

Figure 4 shows the output protonation state of Protonate3D when applied to the 1RO6 structure. Three water molecules were included in the main calculation (each having ∼500 orientation states). The calculation required 60 s to complete on a 2 GHz Pentium IV. The terminal amide of Asn295 was not flipped and the phenol hydrogen of Tyr233 forms a hydrogen bond with the amide oxygen of Asn295. The amide nitrogen is donating one of its hydrogens to the water molecule that is mediating an interaction with His238. Protonate3D assigned an anionic state to His238; a reasonable interpretation of this state is that the N_ε_ of the neutral His238 is singly bonded to the zinc leaving a lone pair on N_δ_. The mediating water is donating one of its hydrogens to form a hydrogen bond with the N_δ_ of His238 and donates its other hydrogen to form a hydrogen bond with a carboxylate oxygen of Asp241. Although one might expect an N_δ_ tautomer for His238, the environment (especially the mediating water) favors the Protonate3D output. It should be kept in mind that the Protonate3D results depend on the 3D coordinates; if the geometry were different (e.g., the water molecules were shifted more toward Ty233 in a different refinement of the crystal structure) then N_δ_ could be predicted protonated.

### Reproduction of peroxidase mechanism

**Figure 5:**
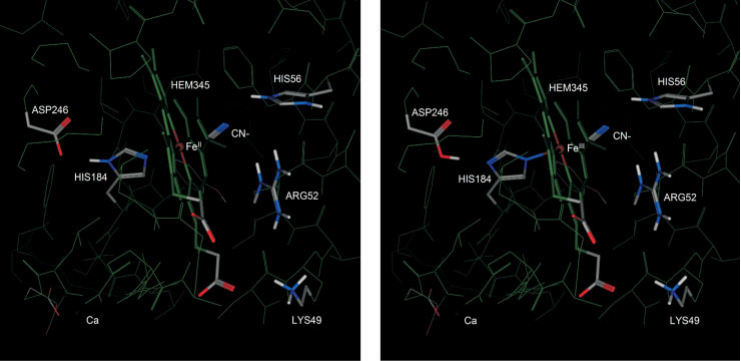
Left: the output of Protonate3D on PDB:1ARU; Asp246 is deprotonated and His184 is protonated corresponding to iron II in the heme. Right: the output of Protonate3D when His184 is constrained to be deprotonated corresponding to iron III in the heme; the result is a protonation of Asp246.

An interesting example of the use of Protonate3D is the reproduction of the activation mechanism surrounding the heme in the peroxidase. PDB:1ARU is a 1.6 Å resolution X-ray crystal structure of a 344 residue peroxidase protein. The active site contains a heme group with a bound cyanide molecule. Waters were deleted and Protonate3D required 30 s to calculate the protonation state of the macromolecule. Figure 5, left, shows the assigned protonation state: His56, Arg52, and Lys49 are all assigned cationic states; the cyanide and two carboxylates on the heme (Hem345) are assigned countering anionic states; His184 is neutral and protonated on N_δ_, which forms a hydrogen bond to the anionic Asp246; the iron in the heme has oxidation number II. Figure 5, right, shows the results of a constrained Protonate3D calculation on the same system. In this constrained case, His184 is constrained to be deprotonated and neutral corresponding to an oxidation number III for the heme iron. Under this constraint, Asp246 was assigned a neutral state by Protonate3D forming a hydrogen bond to the (now) deprotonated N_δ_ of His184. Thus, the constrained calculation neatly reproduces the hydrogen transfer between Asp246 and His184. The speed of Protonate3D is an advantage in these “what if” scenarios. By fixing the protonation state of a group of interest, it is possible to investigate its effects.

### Asp25 in HIV-1 protease

HIV protease features two aspartate residues (Asp25, one in each of monomers of the homodimer) with terminal oxygen atoms ∼3 Å apart whose protonation state is uncertain, depending upon the nature of ligand.^35^ These proximal Asp25 residues provide a challenging case for Protonate3D, because one of the relatively buried Asp25 residues may be protonated. The coordinates for the HIV-1 protease in complex with saquinavir were obtained from PDB:3D1X, a 1.05 Å resolution X-ray crystal structure. The entry consisted of 2 × 99 residues, one saquinavir molecule, one glycerol molecule, 250 water molecules, and three chlorine ions.
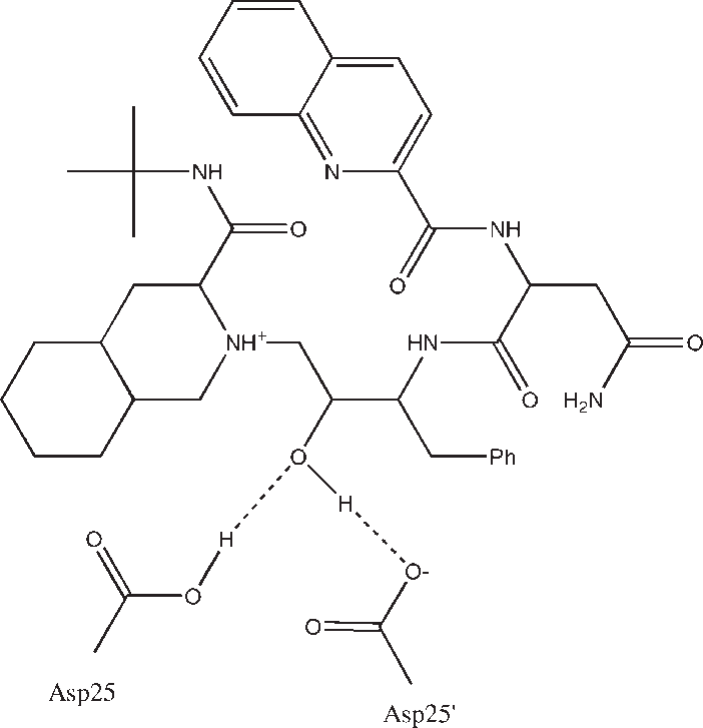


Protonate3D was run at pH 5.0 (the conditions specified in the file); the water molecules were treated as incidental waters (not part of the main optimization). Protonate3D protonated OD1 on Asp25 (chain A) and deprotonated Asp25′ (chain B). In the output configuration, the saquinavir tertiary amine was protonated and saquinavir's hydroxyl donated its hydrogen in a hydrogen bond with OD2 of Asp25′ and accepted the Asp25 carboxylic acid hydrogen in a hydrogen bond (see depiction earlier). This configuration is in agreement with an ab initio and molecular dynamics study showing that the protonated Asp25 is the most stable.^36^

### Glu143 in thermolysin

*Bacillus thermoproteolyticus* thermolysin is a zinc endopeptidase, which catalyses cleavage of peptide bonds. Thermolysin contains a zinc ion in approximately tetrahedral coordination with His143, His146, and Glu166. The catalytic mechanism involves Glu143 that interacts with a zinc-bound water molecule via two hydrogen bonds in the absence of a substrate. A bound substrate undergoes nucleophilic attack from the zinc-bound water.^37^ Glu143 accepts a proton from the zinc-bound water as it attacks the substrate carbonyl carbon; the accepted proton is then transferred to the substrate nitrogen. Many of the interactions involved in the catalysis are exhibited by a bound carbobenzoxy-Phe(P)-Leu-Ala ligand, ZF(P)LA:
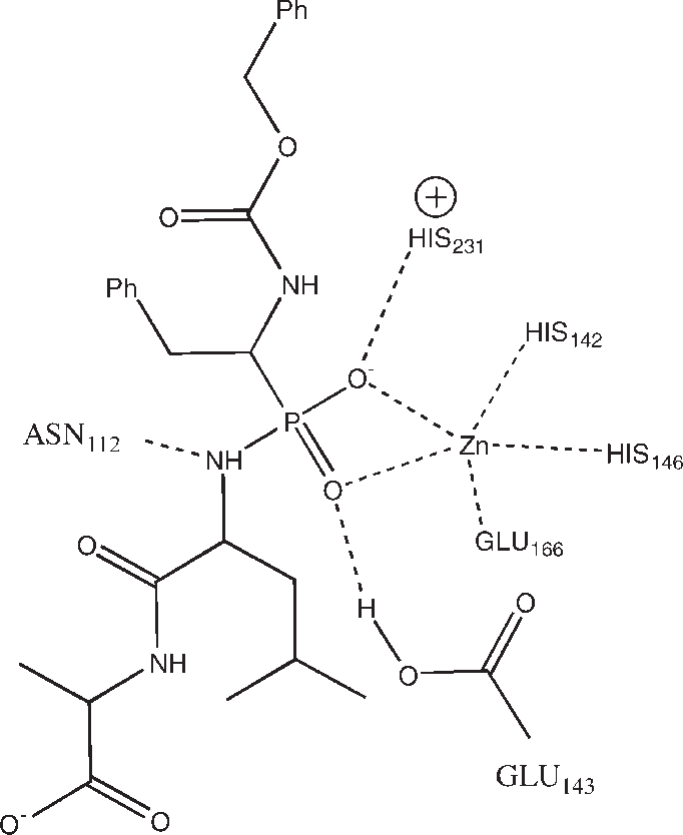


PDB:4TMN is a 1.7 Å resolution X-ray crystal structure of thermolysin complexed with ZF(P)LA, which affords an opportunity to examine Protonate3D's protonation assignment of Glu143. This entry contains a single protein chain of 316 residues, the ZF(P)LA ligand, four calcium ions, one zinc ion, and 162 water molecules. Protonate3D was run at pH 7.0 and the water molecules were omitted. The calculation required 20 s to complete. The results were that OD1 of Glu143 was protonated and formed a hydrogen bond with a phosphate oxygen (see depiction earlier); this protonation appears due to the proximity of an oxygen of Glu143 to an oxygen of the phosphate group. The calculation was repeated but with the ligand omitted and the result was that Glu143 was deprotonated. Thus, the Protonate3D results are consistent with the supposed mechanism of thermolysin.

### Asp30 in nitrophorin 4

The nitrophorins are a family of proteins that use ferric heme to transport nitric oxide from the salivary gland of a blood sucking bug to its victim.^38^ The protonation state of Asp30 exerts crucial influence over surface loop rearrangements related to nitric oxide release.^39^ At pH 5.6, Asp30 is a proton donor for one of two loop hydrogen bonds of the closed form
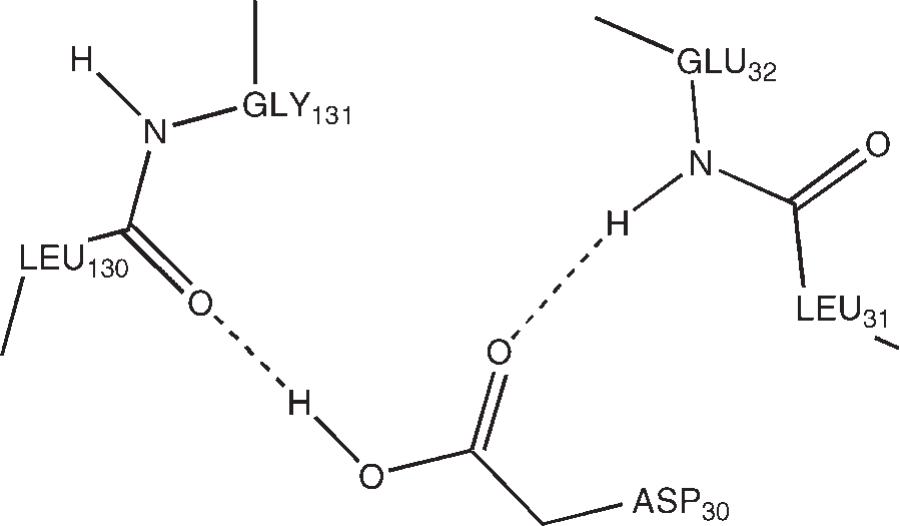
and is in a hydrophobic environment created by Leu31, Pro33, Val36, Leu130, and Leu133. Asp30 in nitrophorin 4 presents an opportunity to test Protonate3D's assignment of protonation state of an aspartate in a buried environment. PDB:1ERX is a 1.4 Å X-ray crystal structure of nitrophorin 4 (184 residues), one heme with a bound nitric oxide and 160 water molecules. The waters were removed and Protonate3D was run at pH 5.6 and required 12 s to complete. The results were that OD2 of Asp30 was protonated, reproducing the hydrogen bond to the backbone carbonyl of Leu130 (see diagram earlier).

### Domain of validity and key limitations

In Protonate3D, different proton configurations are ranked by instantaneous potential energies augmented with a continuum solvation model that is used to estimate both (mean field) solvent interactions and implicit p*K*_a_ shifts of model titratable groups. Key questions naturally arise related to (a) the accuracy of the shifts and consequent ionization state assignments and (b) how the results should be interpreted and what conclusions are legitimate. We will close this Results and Discussion section with an attempt to answer these questions and characterize the accuracy of Protonate3D.

To shed some light on the performance of the Generalized Born solvent model on protein titration problems, we assembled a collection of 908 experimental p*K*_a_ values^40^ for Asp, Glu, His, and Lys residues in 99 proteins with 3D coordinates available in the PDB. Each structure was prepared by deleting waters and group I and group VII counter-ions and running Protonate3D at pH 7. No coordinate refinement was conducted. For each residue (with experimental data), we used Eq. (8) to estimate the p*K*_a_ (with MMFF94 charges, the GB/VI solvent model and ε = 1:80, κ = 0). Now, it must be remembered that this calculation does not (a) take conformational differences into account; (b) sample multiple conformations; (c) take correlated ionizations into account; (d) sample multiple ionization configurations; and (e) consider the possible non-Henderson-Hasselbach behavior of proteins.^41^ Consequently, one cannot expect very accurate estimates of the experimental p*K*_a_; however, we would expect to get a rough trend. In 225 of the cases, the calculated p*K*_a_ values from Eq. (8) produced extreme values (p*K*_a_ values with magnitudes in excess of 20, both positive and negative) corresponding to planar salt bridges, residues near transition metals, buried residues involved in many hydrogen bonds, residues for which coordinate refinement would reduce the magnitude of the estimated p*K*_a_, and residues involved in close contacts. These extreme values are not unexpected because the coordinates of the heavy atoms are fixed and the static microenvironment dominates the estimate. To see this, consider a planar Asp-Arg salt bridge such as
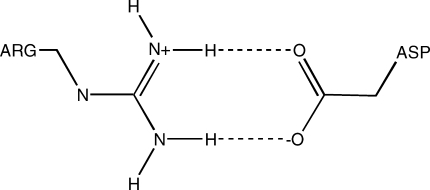


Use of Eq. (8) requires that the energy of protonated form of the Asp be evaluated; in the contemplated configuration, an added proton to one of the oxygen atoms would result in a very unfavorable interaction with the one Arg terminal hydrogen atoms leading to an unrealistic underestimate of the p*K*_a_. In solution, a neutral Asp would likely be farther away from the Arg; in other words, a conformational change is required. We excluded these 225 cases leaving 683 cases for our characterization. The results were a mean absolute error of 3.17 p*K* units and squared correlation coefficient *r*^2^ = 0.47. The errors were skewed in the negative direction by 2.3 p*K* units on average, underestimating the experimental p*K*a values; for the buried residue p*K*a values, the underestimate was 1.6 p*K* units on average. The negative skew is not unexpected because the lack of thermal vibration in Eq. (8) (emphasizing the potential energies of the fixed coordinates) would tend to produce such underestimates. Of course, such a calculation cannot be used for accurate p*K*_a_ estimates but, given the simplicity of the estimate, the results lend some credence to the Protonate3D procedure. A better estimate would likely result if integration over configurations were attempted^42^ or perhaps a statistical correction for the lack of thermal vibrations could be incorporated in the Protonat3D parameter file by adjusting the model compound p*K*a values to reflect the underestimate in an average way. Coulomb potentials, distance dependent dielectrics, and simple pairwise Reaction Field solvent models fare far worse and do not produce any reasonable values (results not shown).

Protonate3D is intended to produce proton coordinates consistent with the given heavy atom coordinates. This is quite different from predicting ionization state in solution and experimental p*K*_a_ (which in many cases are p*K*_1/2_ values), which require thermodynamic consideration of ensembles of conformations and protonation states. The above Asp-Arg salt bridge is an instructive example of how Protonate3D can be expected to perform. The protonation state of the residues participating in the salt bridge is mostly determined by their local environment. The heavy atom coordinates are the dominating factor. Similarly, for the thermolysin example where the proximity of the phosphate anion to Glu143 necessitates some sort of neutralization; Protonate3D is using the model compound p*K*_a_ values, calculated shifts and other interactions to decide what will be neutralized. The Asp246 of peroxidase and Asp25 of HIV-1 protease are similarly rationalized (although in the case of HIV-1 protease, the precise location of the proton needs to be resolved). Asp30 in nitrophorin4 is more challenging in that there are no proximate anions and in this case the solvation model is likely the cause for the successful prediction.

These considerations lead us to believe that Protonate3D will be most successful when attempting to resolve a complex hydrogen bond network, possibly involving, side-chain “flips” and neutralization of unrealistic ionized environments. The static nature of the calculation with its dependence on the given heavy atom (crystal) coordinates means that the assigned ionization states should not be taken necessarily as representative of solution phase ionization states. In addition, borderline cases related to function are probably too subtle for the method. Nevertheless, we expect that the output of Protonate3D will be a good starting point for more sophisticated analyses.

## CONCLUSIONS

We have presented Protonate3D, a method for the automated prediction of hydrogen coordinates given the 3D coordinates of a macromolecular structure. Protonate3D considers side-chain “flip,” rotamer, tautomer, and ionization states of all chemical groups, ligands, and solvent (provided suitable templates are available in a parameter file) during the course of a large scale optimization of the free energy of the system. The energy model includes van der Waals, Coulomb, solvation, rotamer, tautomer, and titration effects. A fast Unary quadratic optimization algorithm including dead-end elimination, mean field theory, and a recursive state search is used to perform the combinatorial optimization without the need for partitioning the system into independent parts based on short interaction cutoffs.

The results of computational experiments on ultra-high resolution X-ray structures suggest that Protonate3D predicts the location of hydrogen atoms to within a 15° dihedral angle with high probability, with the exception of thiol, hydroxyl, and phenol hydrogens where a 37% accuracy rate was suggested. Notwithstanding the 15° dihedral disagreement of the lower accuracy groups with crystal structures, Protonate3D places the hydrogens in these groups in credible low-energy configurations.

Some limitations of the method should be kept in mind. Protonate3D is sensitive to the input 3D coordinates; close contacts and other poor geometry may cause distortions. The default model for transition metals is simplistic—they are treated as isolated ions—and the results should be treated with caution. In addition, the Generalized Born formalism upon which Protonate3D depends may not be suitable for quantitative analysis of weakly acidic or basic groups and, in any event, no single protonation state can be a good representative of such a chemical group.

Protonated3D is intended for use prior to computational procedures such as molecular mechanics, molecular dynamics, crystallographic refinement, ligand–receptor docking, and electrostatic analysis all of which rely on accurate protonation states for best results. Protonate3D is available as part of the Molecular Operating Environment^25^ version 2007.09.

## APPENDIX

Theorem: Let *G* be an *n* by *n* real symmetric matrix and *g* be a real *n*-vector. Let {*I*_1_,…,*I*_*m*_} be an *m*–partition of {1,…,*n*} such that *I*_*i*_ ⊆ {1,…,*n*}, *I*_*i*_ ≠ ø, *I*_*i*_ ∩ *I*_*j*_ = ø if *i* ≠ *j* and ∪{*I*_*i*_} = {1,…,*n*}. For *k* in {1,…,*n*} let *I*(*k*) denote the unique partition member to which *k* belongs. Let β be nonnegative and define

where *e*_*i*_ denotes the vector with a 1 at element *i* and 0 elsewhere. Then, there exists a β_0_ ≥ 0 such that for all β ≤ β_0_, there exists a unique *p* ε (0,1)^*n*^ with the property that *p* = *f*(*p*).

Proof: If *G* = 0, then *f* is constant and *f*(0) is the unique fixed point for all β; hence, taking β_0_ = 1 suffices to prove the theorem. Suppose now, that *G* is nonzero. The derivative of *f* at a point *x* is an *n* by *n* matrix *F*(*x*) with entries *F*_*ab*_(*x*) where

Let *M*(*x*) be the *n* by *n* matrix with entries *M*_*ab*_(*x*) = *f*_*b*_(*x*)δ(*b* ε *I*(*a*)) so that

Now

Thus, ||*F*(*x*)||_∞_ ≤ β ||*G*||_∞_ for all *x*. Let β_0_ = 1/(1 + ||*G*||_∞_) so that for all *x* and for all β ≤ β_0_, we have ||*F*(*x*)||_∞_ ≤ β_0_||*G*||_∞_ < 1. Now, for β ≤ β_0_ and *x*, *y* ε (0,1)^*n*^, there exists (by the Mean Value theorem) ξ such that

Because β_0_||*G*||_∞_ < 1 is constant, by the Banach Fixed Point Theorem there exists a unique point *p* such that *p* = *f*(*p*). QED.
